# Quantification of Ground Reaction Forces During the Follow Through in Trained Male Cricket Fast Bowlers: A Laboratory-Based Study

**DOI:** 10.3390/sports12120316

**Published:** 2024-11-22

**Authors:** Jeffrey Fleming, Corey Perrett, Onesim Melchi, Jodie McClelland, Kane Middleton

**Affiliations:** 1 Sport, Performance, and Nutrition Research Group, School of Allied Health, Human Services and Sport, La Trobe University, Melbourne, VIC 3086, Australia; 2 Kansas City Royals, Kansas City, MO 64129, USA; 3 Physiotherapy, School of Allied Health, Human Services, and Sport, La Trobe University, Melbourne, VIC 3086, Australia

**Keywords:** bowler, delivery stride, impulse

## Abstract

Ground reaction forces (GRFs) are known to be high during front foot contact of fast bowling deliveries in cricket. There is a lack of published data on the GRFs during follow through foot contacts. The aim of this study was to quantify and compare peak GRFs and impulse of the delivery stride and the follow through of fast bowling deliveries. Ten trained male fast bowlers (ball release speed mean ± SD; 32.6 ± 2.3 m/s) competing in the Men’s Victorian Premier League participated in the study. Peak GRF and impulse data were collected using in-ground force plates in a laboratory setting. Linear mixed modelling of GRFs and impulse showed a significant effect of foot strike (*p* < 0.001). Front foot contact had the greatest magnitude of peak vertical GRF (5.569 ± 0.334 BW) but was not significantly greater than back foot recontact (4.471 ± 0.285 BW) (*p* = 0.07). Front foot impact had the greatest vertical impulse (0.408 ± 0.018 BW·s) but was similar to back foot (0.377 ± 0.012 BW·s) and front foot (0.368 ± 0.006 BW·s) recontacts (*p* = 0.070 to 0.928). The high GRF and impulse during the follow through highlights the need for further kinetic and kinematic research on this phase of the fast bowling delivery.

## 1. Introduction

The cricket fast bowling action is an explosive, multi-planar movement that transfers energy accumulated during the run-up to the ball to achieve high ball release speeds, while maintaining accuracy and control [1,2,3,4]. The actions during the delivery stride, which comprises the penultimate and final steps prior to ball release, combine rapid rotation, flexion and lateral flexion of the spine, shoulder counter rotation, and high vertical and anteroposterior (specifically braking) ground reaction forces (GRFs) [5,6]. There has been a large volume of research focused on the fast bowling action but it has done little to reduce the gap in injury rates between fast bowlers and the other positions within cricket [7,8,9,10]. Fast bowlers sustaining injuries at a higher rate is consistent across first-class and academy-level cricketers [8,11,12]. This is especially concerning given the effect injury has on adolescents in the form of increased risk of subsequent injury [13], the inability to continue developing within the sport and earn a professional contract [14], and the impact it has on long-term physical and mental health [13].

While overuse injuries are seen across all stages of athletic development, younger athletes have been found to be at a greater risk [12,13,15,16]. Lumbar bone stress injuries (LBSIs) are typically caused by overuse and are consistently the most prevalent injury in cricket [7,8,12], with a study on professional English fast bowlers reporting an average time lost due to lumbar stress fracture to be between seven and ten months, depending on the injury site [17]. A study on Australian first-class cricketers found that fast bowlers with a history of LBSI were at a greater risk of hamstring, quadriceps, and calf strain when compared with those without a history of LBSI [18]. This further highlights the potential value in understanding and mitigating the risk factors associated with developing LBSI.

The actions involved in fast bowling deliveries are likely to generate significant forces within the body that may contribute to these injuries. The proposed aetiology of bone stress injuries is that the rate of load-induced microdamage outpaces the remodelling of the tissue [19,20]. There is minimal evidence supporting the efficacy of any strategies targeted at aiding and accelerating bone healing [21]; thus, it is crucial to focus efforts on managing the rate of damage in both the prevention and progression of LBSI [19,21,22]. It is widely understood that the risk factors for LBSI are multifactorial [16,20,23], and that bowling in matches and in training is associated with the greatest incidence of LBSI [7,8,9,10]. A study by Alway and colleagues that followed fast bowlers for two years after a biomechanical assessment of bowling technique found no difference in peak GRF at front foot contact (FF1) between those that developed LBSI and those that did not over the study period [24]. Their results added to the growing body of evidence that high GRFs, specific lumbar kinematics, and intrinsic actions of the trunk musculature [5,25] appear to be acting in concert to produce high force on the lumbar vertebrae during the delivery action. In addition, one of the few prospective studies that has investigated the relationship between lumbo-pelvic biomechanics and low back injuries found that those who bowled with greater lumbar loads are at an increased risk of low back injury [26]. Although it is possible that higher ground reaction forces may contribute to higher lumbar loads, for bowlers to develop LBSI, the total magnitude of force experienced by the lumbar vertebrae must be above the threshold level to cause damage and the loading cycles occurring frequently enough to outpace the rate of repair [20].

Likely due to the bowler no longer being able to impart energy to the ball [27], the foot strikes immediately following ball release, i.e., the follow through, have only recently received attention in the literature [28,29]. This is despite evidence suggesting there may be significant and rapid motion occurring [5] while the bowler works to arrest the significant momentum they have generated during the run-up [3,27]. Epifano and colleagues found resultant tibial acceleration was highest during recontact of the foot that was to the back/rear at the time of ball release (back foot recontact; BF2) and that front foot recontact (FF2) had similar magnitude of tibial acceleration to FF1 [28], which has repeatedly been shown to have high GRFs [2,24,30,31,32,33,34,35]. If the GRFs observed during the follow through foot strikes are comparable to those reported at back foot contact (BF1) and FF1, as is suggested by the tibial accelerations [28], these may coincide with significant activation of the paraspinal musculature [5,36] and pose a similar injury risk to the delivery stride [24]. The potential for this phase of the delivery to be contributing a meaningful amount of load-induced microdamage to the lumbar spine warrants further investigation. Therefore, the aim of this study was to quantify and compare the GRFs and impulse from each foot strike in the delivery stride (BF1, FF1) and follow through (BF2, FF2) in trained male cricket fast bowlers. It was hypothesised that peak GRFs would vary across foot strikes (i.e., BF1, FF1, BF2, FF2). Due to the interaction of the force and time components of each foot strike of the delivery and follow through, it was also hypothesised that impulse would vary across the four foot strikes.

## 2. Materials and Methods

As this is the first study to explore the differences in GRFs across the foot strikes of the delivery stride and follow through, it was difficult to estimate a prior effect to use for a sample size calculation. A convenience sample of ten trained [37] male fast bowlers (age: 28.2 ± 4.9 years; height: 185.0 ± 5.4 cm; mass: 87.3 ± 6.9 kg; 1 left-handed; ball release speed 32.6 ± 2.3 m/s [117.4 ± 8.3 km/h]) participated in this study. All participants were competing within the Men’s Victorian Premier League, free from injury, considered a bowler or all-rounder by coaching staff, had more than one year of competitive playing experience, and provided written consent prior to data collection. All procedures were approved by the La Trobe University Ethics Committee (approval number HEC21120).

This observational study required participants to attend the La Trobe University sports biomechanics laboratory on one occasion. A mock cricket pitch was constructed in the laboratory on a 50-m running track covered by 13.5-mm Mondotrack WS (MONDO, Alba, Piedmont, Italy) to allow full run-up and pitch lengths. A set of Kookaburra (Kookaburra Sport, Melbourne, Victoria Australia) spring return stumps were set up along the track to create a regulation pitch length of 20.12 m and white cloth tape was used to mark the bowling and popping creases.

Upon arrival at the laboratory, participant demographics were collected and height and body mass were measured. Participants were then afforded the opportunity to complete a self-directed warmup, which included marking out their full run-up length. Once their warmup was complete, participants were asked to deliver a maximum of 12 over-the-wicket deliveries at a subjective match intensity using a Kookaburra Regulation cricket ball (0.156 kg). Although no batter was present during the testing, right-handed participants were asked to aim at the off stump for a right-handed batter. The left-handed participant was asked to aim at the off stump for a left-handed batter. Ball release speed was measured using a Stalker Pro II radar gun (Stalker Sport, Richardson, TX, USA).

**Figure 1:**
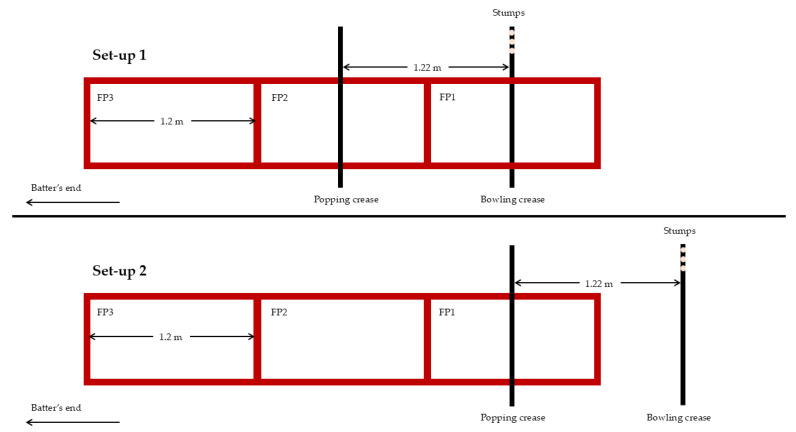
Schematic of testing set-up. Set-up 1 was used to collect data for back foot contact (BF1) on force plate 1 (FP1), front foot contact (FF1) on force plate 2 (FP2), and back foot recontact (BF2) on force plate 3 (FP3). Set-up 2 was used to collect data for front foot contact (FF1) on force plate 1 (FP1), back foot recontact (BF2) on force plate 2 (FP2), and front foot recontact (FF2) on force plate 3 (FP3).

Ground reaction forces at BF1, FF2, BF2, and FF2 were measured using three in-series force plates sampling at 1250 Hz (OR1200600, AMTI, Watertown, MA, USA) that were embedded flush with the laboratory floor using Vicon Nexus software (V2.16, Vicon Motion Systems, Ltd., Oxford, UK). As force was able to be measured across a maximum of three foot strikes per delivery, the testing set-up was changed during the session to allow force from all foot strikes to be measured (Figure 1). The first set-up was constructed so that the bowling-end stumps were placed 15 cm laterally to the force plates and aligned with the middle of the first force plate in the anteroposterior direction, allowing BF1, FF1, and BF2 to be captured. The second set-up was constructed so that the stumps were positioned 1.22 m behind the middle of the first force plate, allowing FF1, BF2, and FF2 to be captured. Participants alternated in delivering their first six deliveries in set-up 1 or set-up 2.

Invalid trials were initially screened during data collection by examining the centre of pressure (CoP) positions in Vicon Nexus. If the foot was not in contact with the correct force plate, or only partially in contact with the correct force plate, the trial was removed and the participant was asked to repeat the trial until there were three successful trials for each of the two set-ups. Participants performed a maximum of six trials in each set-up. Trial data, in the form of .csv files, were exported from Vicon Nexus and imported into MATLAB [38]. CoP data were then further screened, with trials where CoP values were within 0.1 m of the edge of each force plate removed (see Table S3 for the number of trials included in the analysis for each participant). Force data were then filtered using a dual-pass low-pass 2nd order Butterworth filter (cut-off 60 Hz) determined by residual analysis [39].

Peak GRF values were calculated using the ‘findpeaks’ MATLAB function, which was employed separately for vertical and braking GRFs. To calculate impulse, the time between the start (when vertical force exceeded 25 N for the first time prior to peak GRF) and end (when vertical force dropped below 25 N for the first time after peak vertical GRF) of each foot strike was calculated. These times were then multiplied by the mean force to calculate vertical and braking impulse. Once GRF and impulse values had been calculated for all valid foot strikes, data were exported to a .csv file for further analyses.

The .csv datafile was imported into the R Statistical package [40]. The data were normalised to participant bodyweight (BW) to convert from GRF in Newtons (N) to BW and impulse in Newton-seconds (N**·**s) to bodyweight-seconds (BW**·**s), respectively. The ‘lmerTest’ package in R [41] was used to fit linear mixed models to examine the effect of foot strike on the normalised GRF and normalised impulse, respectively. Given each participant had repeated measurements, data from each bowling trial were entered into the model. Various configurations of models were assessed; the models that had the lowest Bayesian Information Criterion were chosen for further analysis. Model diagnostics, including residual plots, checks for normality, and influential observations revealed no violations of model assumptions for LMMs. The final models had foot strike as a fixed effect, with participant as a random intercept and foot strike as a random slope. This allowed for individual variability in baseline levels of GRF or impulse and for the effect of foot strike to vary across participants. The model formulas were specified as follows:

$$y\sim foot\;strike+\left(1+foot\;strike\;\right|\;participant)$$

where y is the dependent variable, ‘1’ specifies a random intercept, the first ‘foot strike’ is the fixed factor, the second ‘foot strike’ is the random slope, and ‘participant’ is the random factor.

Estimated marginal means (EMMs) were calculated for each of the dependent variables using the ‘emmeans’ package in R [42] to then conduct *post-hoc* pairwise comparisons to assess for differences between each level of foot strike. The EMMs were calculated using the same model structure as the LMMs, where foot strike was specified as a fixed effect and participant as a random intercept, with foot strike as a random slope. This model structure effectively averaged the dependent variable values over random participant effects, isolating the group-level effect of foot strike while holding participant differences constant. The random intercept and slope model structure allowed for the modeling of within participant variability while also ensuring each participant’s data contributed appropriately to the group estimate. This approach allowed for the isolation and interpretation the foot strike fixed effect had on the dependent variable. The comparisons were assessed with an α of 0.05 using Tukey’s adjustment to control for family-wise error rates.

## 3. Results

### 3.1. Ground Reaction Force

**Figure 2:**
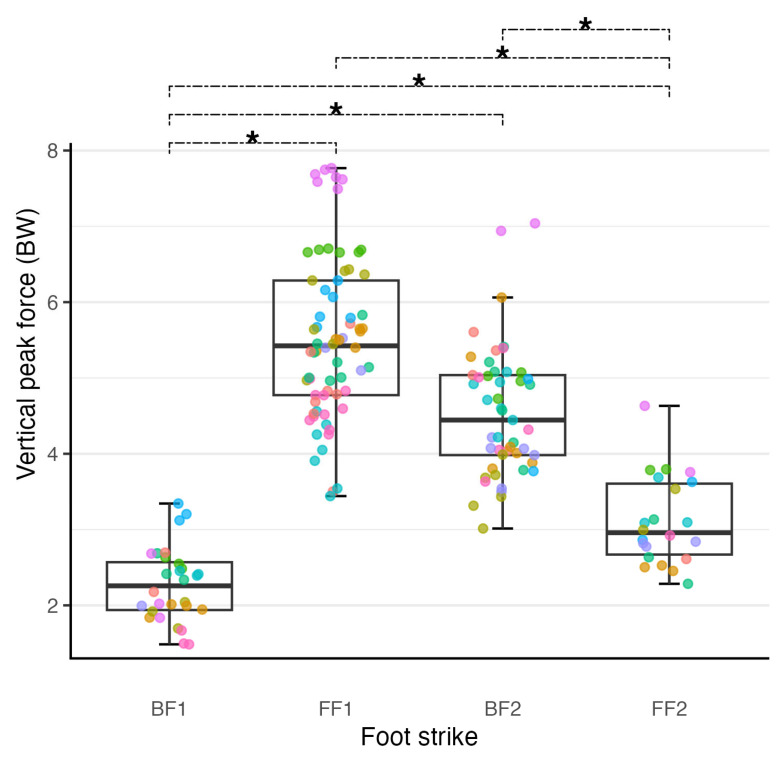
Scatter plot combined with a box plot to illustrate the distribution of the vertical ground reaction force (GRF) data collected at each foot strike. Scatter plot points are colour-coded by participant to aid in visualising both intra-participant and inter-participant variability of the data. Colour coding of participants is consistent across figures. The full width line represents the median of the data. The upper and lower hinges of the boxes represent the first and third quartiles of data, respectively, the whiskers extending to the largest or smallest value no more than 1.5 times the distance between the first and third quartile. To aid in discerning individual data points, a random amount of noise has been added to the x value for each scatter plot point; the y values are unaltered. * Denotes estimated marginal mean of foot strikes are different to at least the 0.05 level.

There were significant main effects of foot strike on peak vertical GRF (F(3, 10.057) = 46.676, *p* < 0.001) and peak braking GRF (F(3, 10.155) = 21.125, *p* < 0.001) (Table 1). The largest magnitude of peak vertical GRF was observed at FF1, followed by BF2 then FF2 and BF1. All paired comparisons (Table S1) were significantly different (*p* < 0.008) except between FF1 and BF2, which was not significantly different (*p* = 0.070) (Figure 2). FF1 also had the largest braking GRF and was significantly greater than all other foot strikes (*p* < 0.004). All other pairwise comparisons for braking GRF were not significantly different (*p* = 0.334 to 0.971).

### 3.2. Impulse

**Figure 3:**
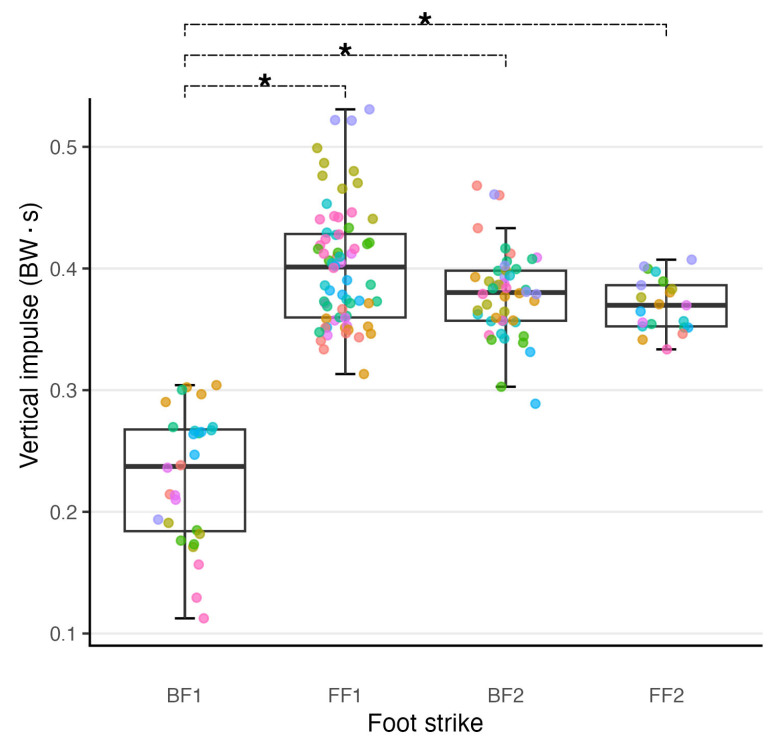
Scatter plot combined with a box plot to illustrate the distribution of the vertical impulse data collected at each foot strike. Scatter plot points are colour-coded by participant to aid in visualising both intra-participant and inter-participant variability of the data. Colour coding of participants is consistent across figures. The full width line represents the median of the data. The upper and lower hinges of the boxes represent the first and third quartiles of data, respectively, the whiskers extending to the largest or smallest value no more than 1.5 times the distance between the first and third quartile. To aid in discerning individual data points a random amount of noise has been added to the x value for each scatter plot point, the y values are unaltered. * Denotes estimated marginal mean of foot strikes are different to at least the 0.05 level.

There were significant main effects of foot strike on vertical impulse (F(3, 9.761) = 23.605, *p* < 0.001) and braking impulse (F(3, 11.803) = 14.749, *p* < 0.001) (Table 2). BF1 had the lowest vertical impulse and was significantly different from all other foot strikes (*p* < 0.001) (Figure 3). All other foot strike pairwise comparisons were not significantly different (*p* = 0.070 to 0.928) (Table S2). FF2 recorded the lowest braking impulse and was significantly lower than FF1 (*p* = 0.014) and BF1 (*p* = 0.001). All other foot strike pairwise comparisons for braking impulse were not significantly different (*p* = 0.168 to 0.994).

## 4. Discussion

This study quantified and compared the peak vertical and braking GRFs and vertical and braking impulse of both the foot strikes of the delivery stride (BF1, FF1) and follow through (BF2, FF2) in trained male fast bowlers. Our hypothesis that peak GRF would vary across foot strikes was supported, as was our hypothesis that due to the interaction of the force and time components of each foot strike of the delivery and follow through, impulse would also vary across the four foot strikes. Vertical GRF and impulse values followed a similar structure, with the greatest magnitudes being at FF1 followed by BF2, then FF2 and finally BF1. Peak braking force and impulse had slightly differing patterns. FF1 had the greatest braking GRF with all other foot strikes being smaller, but similar to each other. Braking impulses were largely similar with only FF2 being significantly lower than BF1 and FF1.

The vertical GRF values for BF1 and FF1 found in this study are in line with those reported previously in the literature [2,24,31,33,34,35,43]. The braking GRF and impulse were slightly lower but still within the typical ranges when compared to other published data [43]. The high vertical GRF and impulse found in this study at BF2 is a novel and important addition to the understanding of fast bowling from a kinetic viewpoint. The peak vertical GRF values at BF2 in this study are as high and in some cases higher than those reported at FF1 by Sennington and colleagues [43], with the vertical GRF at FF2 also being higher than reported values for BF1 [31]. The relatively large GRF at BF2 is likely due to the requirement of bowlers to decelerate their centre of mass in the follow through, due to the linear momentum developed in the run-up, with the back leg accelerating ahead of the centre of mass and contacting the ground. The majority of studies that report GRF or impulse values at foot strikes almost exclusively report FF1, with the exception of a few studies also reporting BF1 values [31,44]. This is understandable when the focus of the research is the kinetic, kinematic, or technical aspects of bowling that contribute to ball release speed, given the bowler has no effect on the ball post release, which occurs during FF1. However, this limited reporting of kinetic and kinematic data on the follow through presents a risk that important information is overlooked when it comes to trying to understand the effect fast bowling has on the body for the purposes of injury prevention and management.

To date, the only other published research with any specific data on BF2 and FF2 was by Epifano and colleagues [28,29]. Their findings showed BF2 had the highest tibial acceleration followed by FF2, then FF1 and BF1. However, the similar peak vertical GRF between FF1 and BF2 in the current study confirms Epifano and colleagues’ assertion that tibial accelerations cannot be used as a surrogate for GRFs or impulse during foot strikes. GRF represents only part of the load on internal structures as has been demonstrated by segmental modelling of spinal segments [5,25] and on the tibia [45] in running. As such, the independent effect GRF may have on injury is not completely clear [24]. A potential confounder of any effect that GRF may have had on prospective LBSI injury reported by Alway and colleagues is that their study limited data collection to the period of time between BF1 and ball release. Based on the results of this study, the GRF occurring during the follow through foot strikes may produce high lumbar loads, which have been shown to occur during the delivery stride and associated with LBSI [26]. Further research is required to quantify lumbar kinematics and kinetics during the follow through to determine if high lumbar loads are experienced during this phase of the delivery action.

Our hypothesis that foot strike would significantly affect impulse was supported; however, the differences were not as pronounced as those seen in GRF. The relative similarity of the vertical impulse experienced across FF1, BF2, and FF2 is a particularly interesting finding of this study. Even though the GRF reduces across these foot strikes, the similarity in impulse indicates that the GRF remains relatively high and the foot contact time increases. The high vertical impulse could have implications for the role of the follow through foot strikes regarding the development of LBSI, as the duration over which a force is exerted is an important factor in the resulting strain of the bone [19]. The GRF represents an extrinsic applied force and is added to the intrinsic force from contracting muscles acting on the lumbar vertebrae [25]. Lumbar spinal segment modelling of the period from BF1 to ball release by Ferdinands et al. [5] suggests the trunk musculature may be working during the follow through to control the high segmental angular velocity seen during FF1. Strain damage of bones will occur when the sum of forces is above the damage threshold regardless of the independent contributions of the extrinsic or intrinsic factors [20]. This longer duration of relatively high force during the follow through means there is an increased window over which the sum of the forces may exceed the damage threshold for lumbar vertebrae. However, it is equally plausible that the force may be below the damage threshold, and the longer duration of the follow through foot strikes may have a negligible influence on the development of LBSI. Without quantifying lumbar forces and the ability to compare to a clinical threshold, the casual relationship between impulse and LBSI remains speculative. Further research should explore links between these findings and lumbar injuries.

This study presents valuable additions to the current understanding of the kinetic aspects of fast bowling, particularly the follow through foot strikes; however, there are some limitations which the authors acknowledge. Although adequate total space was provided, laboratory conditions are not the same as typical match or training conditions. The synthetic running track as opposed to a grass wicket and the requirement for the bowler to land on the force plates when bowling present departures from in situ fast bowling. This may have resulted in lower GRFs than would be experienced in typical match or training conditions. These laboratory accommodations are a concession that must be made until there is a valid and reliable way to collect high-quality GRF data on a representative wicket. The ten participants in this study were adult males participating at a state premier league level. As such we caution the generalisation of the results to amateur or elite male populations as there are documented differences in GRFs between levels of competitors [46]. While kinetic data on female fast bowlers are sparse, Felton and colleagues did find differences in kinematic data between males and females, particularly in centre of mass horizontal velocity at BF1 and vertical velocity at FF1 [47], which may suggest differences in GRFs at the foot strikes and future research should quantify the GRFs across the foot strikes of the delivery stride and follow through in female fast bowlers. The sample size may have reduced the statistical power to detect small effects and led to reduced precision of effect sizes. However, the use of linear mixed-effects models are robust to small sample sizes by accounting for individual variability and can mitigate biases introduced by small sample sizes by appropriately modelling with-in participant correlations. The authors believe the aforementioned limitations do not detract from the findings of this study and encourage future confirmatory studies to validate these findings in larger sample sizes of elite male and female fast bowlers.

## 5. Conclusions

This study quantified and compared GRFs and impulse during the foot strikes of both the delivery stride (BF1 and FF1) and the follow through (BF2, FF2) of fast bowling. Foot strike was found to have a significant effect on both GRF and impulse along both the vertical and braking directions. FF1 had the greatest vertical GRF but it was not significantly different to BF2. Vertical impulse was highest at FF1 but was not significantly different to the impulse at BF2 and FF2. This is the first study to publish GRF and impulse data for the foot strikes during the follow through of fast bowling. Our findings show that there are high GRFs and impulse being experienced by the bowler during the follow through, which may have implications for performance and injury. However, further research is recommended to fully quantify kinematics and kinetics (e.g., lumbar joint moments) during the follow through to understanding how this phase of the delivery may relate to injury, particularly LBSI.

## Figures and Tables

**Table 1:** Ground reaction force (GRF) values at each foot strike. Results presented are estimated marginal means (EMM) ± standard error (SE) and 95% confidence intervals (CI). Results of the Type III ANOVA for the LMMs, with the effect size calculated using partial epsilon squared ($\varepsilon^{2})$.

			ANOVA			
	EMM ± SE	95% CI	Degrees of Freedom	*F* Statistic	*p* Value	$\varepsilon^{2}$
Peak vertical force (BW)			3, 10.057	46.676	<0.001	0.91
BF1 ^abc^	2.271 ± 0.152	1.935–2.606				
FF1 ^ae^	5.569 ± 0.334	4.835–6.304				
BF2 ^bf^	4.471 ± 0.285	4.085–5.336				
FF2 ^cef^	3.127 ± 0.179	2.733–3.522				
Peak braking force (BW)			3, 10.155	21.125	<0.001	0.81
BF1 ^a^	1.112 ± 0.129	0.827–1.396				
FF1 ^ade^	1.763 ± 0.041	1.673–1.852				
BF2 ^d^	1.175 ± 0.120	0.912–1.439				
FF2 ^e^	0.995 ± 0.110	0.713–1.196				

a = difference between BF1 and FF1; b = difference between BF1 and BF2; c = difference between BF1 and FF2; d = difference between FF1 and BF2; e = difference between FF1 and FF2; f = difference between BF2 and FF2. *p* < 0.05 for all marked differences.

**Table 2:** Impulse values at each foot strike. Results presented are estimated marginal means (EMM) ± standard error (SE) and 95% confidence intervals (CI). Results of the Type III ANOVA for the LMMs, effect size calculated using partial epsilon squared ($\varepsilon^{2})$.

			ANOVA			
	EMM ± SE	95% CI	Degrees of Freedom	*F* Statistic	*p* Value	$\varepsilon^{2}$
Vertical impulse (BW·s)			3, 9.761	23.605	<0.001	0.01
BF1 ^abc^	0.223 ± 0.017	0.186–0.260				
FF1 ^a^	0.408 ± 0.018	0.369–0.447				
BF2 ^b^	0.377 ± 0.012	0.351–0.402				
FF2 ^c^	0.368 ± 0.006	0.354–0.382				
Braking impulse (BW·s)			3, 11.803	14.749	<0.001	0.01
BF1 ^c^	0.039 ± 0.007	0.024–0.054				
FF1 ^e^	0.042 ± 0.008	0.024–0.060				
BF2 ^d^	0.017 ± 0.009	−0.003–0.037				
FF2 ^ce^	−0.003 ± 0.005	−0.013–0.008				

a = difference between BF1 and FF1; b = difference between BF1 and BF2; c = difference between BF1 and FF2; d = difference between FF1 and BF2; e = difference between FF1 and FF2 *p* < 0.05 for all marked differences.

## Data Availability

The original contributions presented in the study are included in the article; further inquiries can be directed to the corresponding author(s).

## Supplementary Materials

The following supporting information can be downloaded at: https://www.mdpi.com/article/10.3390/sports12120316/s1, Table S1: GRF paired comparisons; Table S2: Impulse paired comparisons; Table S3: Participant trials and data points.
