# Supplementary material for: Quantification of Ground Reaction Forces During the Follow Through in Trained Male Cricket Fast Bowlers: A Laboratory-Based Study
Source: Sports (Basel). 2024 Nov 22;12(12):316. doi: 10.3390/sports12120316 (PMC11680029; doi:10.3390/sports12120316)
Supplement: Supplementary file 1 [file sports-12-00316-s001.zip › sports-3284027-supplementary.pdf]

Supplementary Table S1. GRF paired comparisons. Differences between the estimated marginal means of each foot strike pair.

Contrast Pair	Mean Difference Estimate ± SE	95% CI	df	t	*p*
**Vertical GRF**					
BF1 - FF1	-3.299 ± 0.345	-4.335, -2.262	11.1	-9.565	<0.001
BF1 - BF2	-2.44 ± 0.305	-3.356, -1.523	11.2	-7.992	<0.001
BF1 - FF2	-0.857 ± 0.208	-1.482, -0.231	11	-4.124	0.008
FF1 - BF2	0.859 ± 0.307	-0.063, 1.781	11.1	2.8	0.070
FF1 - FF2	2.442 ± 0.259	1.662, 3.222	10.9	9.442	<0.001
BF2 - FF2	1.583 ± 0.234	0.88, 2.286	11.1	6.769	<0.001
**Breaking GRF**					
BF1 - FF1	-0.651 ± 0.125	-1.027, -0.275	11.1	-5.204	0.001
BF1 - BF2	-0.064 ± 0.147	-0.507, 0.379	11.1	-0.433	0.971
BF1 - FF2	0.157 ± 0.091	-0.118, 0.432	11	1.717	0.361
FF1 - BF2	0.587 ± 0.13	0.197, 0.978	11.1	4.521	0.004
FF1 - FF2	0.808 ± 0.108	0.482, 1.134	11.1	7.45	<0.001
BF2 - FF2	0.221 ± 0.124	-0.154, 0.595	11	1.776	0.334

Supplementary Table S2. Impulse paired comparisons. Differences between the estimated marginal means of each foot strike pair.

Contrast Pair	Mean Difference Estimate ± SE	95% CI	df	t	*p*
**Vertical Impulse**					
BF1 - FF1	-0.185 ± 0.031	-0.278, -0.092	11.1	-5.962	<0.001
BF1 - BF2	-0.153 ± 0.02	-0.215, -0.092	11.1	-7.517	<0.001
BF1 - FF2	-0.145 ± 0.019	-0.203, -0.086	10.9	-7.441	<0.001
FF1 - BF2	0.031 ± 0.022	-0.034, 0.096	11.2	1.446	0.499
FF1 - FF2	0.04 ± 0.014	-0.003, 0.083	10.9	2.81	0.070
BF2 - FF2	0.009 ± 0.014	-0.035, 0.052	11	0.605	0.928
**Breaking Impulse**					
BF1 - FF1	-0.003 ± 0.012	-0.04, 0.033	11.1	-0.255	0.994
BF1 - BF2	0.022 ± 0.014	-0.02, 0.064	11.1	1.578	0.428
BF1 - FF2	0.041 ± 0.008	0.018, 0.065	10.5	5.335	0.001
FF1 - BF2	0.025 ± 0.011	-0.008, 0.058	11.1	2.256	0.168
FF1 - FF2	0.045 ± 0.012	0.009, 0.08	10.9	3.792	0.014
BF2 - FF2	0.02 ± 0.009	-0.009, 0.048	10.6	2.062	0.227

Supplementary Table S3. Participant trials and data points.

Participant	Trials included	Included data points			
		BF1	FF1	BF2	FF2
P01	7	2	7	4	1
P02	7	4	7	6	3
P03	7	3	7	6	2
P04	6	3	6	4	2
P05	8	3	8	7	3 (2)*
P06	7	3	7	7	3
P07	6	3	6	2	2
P08	7	1	3	6	3
P09	8	3	7	2	2
P10	10	3	10	5 (4)*	1

* During the analysis of the centre of pressure in it was found that the centre of pressure for two trials transitioned off the force plates after a valid peak GRF value was obtained. The peak GRF values for these trials was included in the analysis but the impulse data for these trials was removed from the analysis.
